# The Predictive Role of Raw Bioelectrical Impedance Parameters in Water Compartments and Fluid Distribution Assessed by Dilution Techniques in Athletes

**DOI:** 10.3390/ijerph17030759

**Published:** 2020-01-24

**Authors:** Ruben Francisco, Catarina N. Matias, Diana A. Santos, Francesco Campa, Claudia S. Minderico, Paulo Rocha, Steven B. Heymsfield, Henry Lukaski, Luís B. Sardinha, Analiza M. Silva

**Affiliations:** 1 Exercise and Health Laboratory, CIPER, Faculdade Motricidade Humana, Universidade de Lisboa, 1499-002 Lisbon, Portugal; ruben92francisco@gmail.com (R.F.); cmatias@fmh.ulisboa.pt (C.N.M.); dianasantos@fmh.utl.pt (D.A.S.); cminderico@gmail.com (C.S.M.); procha@fmh.ulisboa.pt (P.R.); lbsardinha55@gmail.com (L.B.S.); analiza.monica@gmail.com (A.M.S.); 2 Departments of Biomedical and Neuromotor Sciences, University of Bologna, 40121 Bologna, Italy; 3 Department for Life Quality Studies, University of Bologna, 47921 Rimini, Italy; 4 Pennington Biomedical Research Foundation, Baton Rouge, Louisiana, LO 70808, USA; Steven.Heymsfield@pbrc.edu; 5 Department of Kinesiology and Public Health Education, Hyslop Sports Center, University of North Dakota, Grand Forks, ND 58202, USA; henry.lukaski@und.edu

**Keywords:** phase angle, resistance, reactance, bioimpedance, health

## Abstract

The aims of this study were to analyze the usefulness of raw bioelectrical impedance (BI) parameters in assessing water compartments and fluid distribution in athletes. A total of 202 men and 71 female athletes were analyzed. Total body water (TBW) and extracellular water (ECW) were determined by dilution techniques, while intracellular water (ICW) was calculated. Fluid distribution was calculated as the ECW/ICW ratio (E:I). Phase angle (PhA), resistance (R) and reactance (Xc) were obtained through BI spectroscopy using frequency 50kHz. Fat (FM) and fat-free mass (FFM) were assessed by dual-energy X-ray absorptiometry. After adjusting for height, FM, FFM, age and sports category we observed that: PhA predicted ICW (females: β = 1.62, *p* < 0.01; males: β = 2.70, *p* < 0.01) and E:I (males and females: β = −0.08; *p* < 0.01); R explained TBW (females: β = −0.03; *p* < 0.01; males: β = −0.06; *p* < 0.01) and ECW (females: β = –0.02, *p* < 0.01; males: β = −0.03, *p* < 0.01) and ICW (females: β = –0.01, *p* < 0.053; males: β = –0.03 *p* < 0.01); and Xc predicted ECW (females: β = −0.06, *p* < 0.01; males: β = −0.12, *p* < 0.01). A higher PhA is a good predictor of a larger ICW pool and a lower E:I, regardless of body composition, age, height, and sports category. Lower R is associated with higher water pools whereas ECW expansion is explained by lower Xc. Raw BI parameters are useful predictors of total and extracellular pools, cellular hydration and fluid distribution in athletes.

## 1. Introduction

Assessing fluid balance to monitor the hydration status in athletes has received substantial interest over the last years for maximizing performance [1,2,3]. 

Bioelectrical impedance analysis (BIA) has been widely used as a rapid, safe and non-invasive method for monitoring active adults performing recreational exercise and elite athletes [4,5,6] and in estimating body composition and nutritional status in healthy non-athletes and adults with clinical conditions [7,8,9]. Alternating current is introduced into the body by bioimpedance electronic devices at single or multiple frequencies. Passive bioelectrical measurements can be related to physiological or body composition parameters.

In addition, this test is important because it enables the use of resistance (R), reactance (Xc) and phase angle (PhA) as indices of biological variables. The resistance (R) arises from body fluids, i.e., extra and intracellular fluids behave as resistive components and resistance is inversely proportional to fluid volume and Reactance (Xc) arises from cell membranes [8]. PhA has been studied as an indicator of nutritional status and disease prognosis, mortality and cellular vitality [10,11]. Its assessment may be valuable for coaches, physicians, nutritionists, and exercise physiologists to provide specific recommendations to improve athletic performance and to avoid compromising health status. 

For example, from the wide range of conditioning factors of an athlete’s career, muscle injuries can be decisive in limiting participation in training or competitions. Muscle injuries cause marked reductions in R, Xc and PhA [12]; these changes indicated cell membrane disruption and may suggest alteration of fluid compartments. Further, in disease status, the PhA is sensitive to electrical changes in tissues [13]. In a recent study the authors verified that PhA was negatively correlated with fluid retention evaluated using the extracellular water (ECW): total body water (TBW) ratio in patients with advanced cancer [14]. Bioimpedance analysis also has been the focus of previous studies involving patients with hemodialysis [15] and acute heart failure [16].

Thus, because of its relevance, it is pertinent to understand the role of PhA and raw bioelectrical impedance (BI) parameters on water compartments and fluid distribution for potentially provide strategies to promote an adequate fluid balance and cellular hydration.

To our knowledge, only one study analyzed the relation between raw BI variables and fluid compartments in athletes [17]. Marini and colleagues [17] observed that higher values of PhA were related to lower values of the E:I ratio, commonly used as an indicator of fluid distribution. However, the authors did not explore the magnitude of the correlation between raw BI parameters and the water compartments considering the potential impact of body composition. Indeed, the PhA is associated with fat free mass (FFM) and fat mass (FM) and therefore, these variables should be accounted for when exploring the association between this BI marker and fluid-related compartments [10]. However, this analysis is yet to be determined. 

Moreover, simple methods are required to assess water compartments and fluid distribution in athletes. Raw BI measure may provide relevant information related to reference methods, i.e., dilution techniques. Therefore, the aim of this study was to investigate the usefulness of raw bioelectrical impedance spectroscopy (BIS) parameters (R, Xc and PhA) at frequency 50kHz on water compartments and fluid distribution, assessed through dilution techniques, in athletes, adjusting for the potential effect of confounding variables such as height, age, body composition, and sports category.

## 2. Material and Methods

### 2.1. Participants

A total of 202 males (21.5 ± 4.5y) and 71 females (20.4 ± 5.2y) participated in this observational cross-sectional study. The inclusion criteria were: 1) Tanner stage V or greater [18]; 2) >10 hours of sport specific training per week; 3) have negative anti-doping results, and 4) currently not taking any medication or dietary supplements. Prior to participation, all athletes (parental or guardian) gave their written informed consent, with all procedures approved by the Ethics Committee of the Faculty of Human Kinetics, Technical University of Lisbon and conducted in accordance with the declaration of Helsinki

### 2.2. Anthropometric Measurements

Height and weight were measured while the subject wore a swimsuit and standardized procedures were used [19]. 

### 2.3. Fat Mass (FM) and Fat-Free Nass (FFM)

Whole body FFM and FM were determined by dual-energy X-ray absorptiometry (Hologic Explorer W, QDR for Windows version 12.4, Waltham, MA, USA). In our laboratory, in ten healthy adults, the test-retest CV for FFM and FM are 0.8% and 1.7%, respectively. The technical error of measurement (t.e.m.) is 0.4kg. 

### 2.4. Hydration Status

After a fasting baseline urine sample was collected, the specific gravity (USG) was determined using a refractometer (Urisys 1100, Roche Diagnostics, Portugal) to ensure that all athletes were euhydrated (USG < 1.010) [20]. The coefficient of variation (CV) of the urine specific gravity procedure in our laboratory based on 10 adults is 0.1% [21].

### 2.5. Total Body Water

Total body water (TBW) was measured by deuterium dilution using a Hydra stable isotope ratio mass spectrometer (PDZ, Europa Scientific, UK). After a 12h fast, the first urine sample was collected. Each participant was given an oral dose of 0.1g of 99.9% ^2^H_2_O per kg of body weight (Sigma-Aldrich; St. Louis, MO). After a 4 h equilibration period, during which no food or beverage was consumed, a urine sample was collected. Urine and diluted dose samples were prepared for ^1^H/^2^H analysis using the equilibration technique of Prosser and Scrimgeour [22], using procedures described elsewhere [23]. The CV based on 10 repeated measures for TBW with the stable isotope ratio mass spectrometry in this laboratory is 0.3% [21].

### 2.6. Extracellular Water

Through dilution of sodium bromide (NaBr) was determined extracellular water (ECW). After collection of a saliva sample, each participant was asked to drink 0.030 g of 99.0% NaBr (Sigma-Aldrich; St. Louis, MO) per kg of body weight, diluted in 50 mL of distilled-deionized water, using procedures described elsewhere [23]. Saliva samples were collected into salivettes. Then, the samples were centrifuged and frozen for posterior analyses. The CV in our laboratory based on 10 repeated measures for ECW using high-performance liquid chromatography is 0.4% [23].

### 2.7. Intracellular Water

Intracellular water (ICW) was determined as the difference between TBW and ECW using the dilution techniques (ICW = TBW − ECW).

### 2.8. Raw BIA Parameters

Whole body PhA, R and Xc were obtained using bioelectrical impedance spectroscopy (BIS) analyzer (model 4200, Xitron Technologies, San Diego, CA) at frequency of 50kHz. Measurements were performed after a 10-minute period of rest with the participant in a supine position with a leg opening of 45° compared to the median line of the body and the upper limbs positioned 30° away from trunk. Four electrodes were placed on the dorsal surfaces of right foot and ankle and right wrist and hand, as described elsewhere [24]. The biological reliability determined in six participants in our laboratory for R and Xc at 50kHz was 0.6 and 1.5%, respectively [25]. 

### 2.9. Statistical Analysis

Descriptive analysis including means ± standard deviations were calculated. Normality was evaluated using Shapiro–Wilk test. Since the data showed a normal distribution a person’s correlation was performed between independent (PhA, R and Xc), and dependent variables (ICW, ECW, TBW and E:I ratio) and an independent sample *t* test was used to compare males and females. Multiple regression analysis was used to assess the association between each of the dependent variables and each independent variable using the unstandardized beta coefficient and adjusting for the confounding variables height, sports category, FFM, FM and age. The variable “sports category” was created to differentiate athletes from modalities of (1) ‘endurance’ where athletes of swimming, pentathlon, triathlon and sailing were included, (2) ‘velocity/power’ that encompassed modalities of judo, karate, taekwondo and athletics and (3) ‘team sports’ which included basketball, handball, volleyball, rugby and soccer. 

Significance level was set at *P* < 0.05. The data was analyzed with IBM SPSS Statistics program (TM 24.0 for MacOS). If more than one variable was a predictor in the model, a calculated to evaluate multicollinearity, and values below five were considered not to have multicollinearity issues.

## 3. Results

Table 1 shows the main characteristics of the athletes included in the sample and identifies the differences between males and females.

Body mass and height were higher in the male group (*p* < 0.01). Male sample present higher values of FFM (*p* < 0.01). Whereas the female sample presents FM values significantly greater than the males (*p* < 0.01). R, Xc, R/H and Xc/H were higher in the female group (*p* < 0.01), while the PhA was higher in the male group (*p* < 0.01). Men presented higher ICW, ECW and TBW values (*p* < 0.01), while women had higher E:I ratio values (*p* < 0.01).

The association between the raw BIA parameters and the water compartments (ICW, ECW and TBW) and fluid distribution (E:I ratio) is presented in Table 2.

For PhA, the strongest correlation was shown with E:I ratio. The PhA was inversely related with E:I ratio in females (*r* = −0.52, *p* < 0.01) and males (*r* = −0.55, *p* < 0.01). While R/H was negatively associated with TBW (*r* = −0.84, *p* < 0.01 for females and *r* = −0.87, *p* < 0.01 for males). For Xc, an association was found with ECW (*r* = −0.76, *p* < 0.01 for females and *r* = −0.68, *p* < 0.01 for males). Additionally, for FFM we observed the strongest correlation with TBW for males (*r* = 0.91, *p* < 0.01) and females (*r* = 0.92, *p* < 0.01). For FM, the strongest correlation was observed with TBW for men (*r* = 0.64, *p* < 0.01) and with ECW in women (*r* = 0.55, *p* < 0.01).

As described in Table 3, the PhA was a significant predictor of ICW for both sexes (β = 1.62; *p* < 0.01 for females and β = 2.70; *p* < 0.01 for males), regardless of height, sports category, FFM, FM and age. PhA explained alone (without adjustment to covariables) 12% and 17% of the ICW values, respectively for females and males.

PhA was also considered a significant predictor of TBW in males (β = 2.01; *p* < 0.01), explaining alone about 9% of TBW values. In both sexes, PhA explained 27% and 30% of E:I, respectively. When adjusted to covariables, the PhA presented a negative β value (β = −0.08; *p* < 0.01 for both), which means that it is inversely related to the fluid distribution in females and males.

R explains alone (the unadjusted r^2^, i.e., the predictive power of R in explaining the variability of the ICW values without adjusting for confounding variables) about 33% and 45% of the ICW values in females and males, respectively. When adjusted to covariables (Table 4) the R is inversely related to the ICW for females (β = −0.01, *p* = 0.053) and males (β = −0.03, *p* < 0.01).

In both sexes, R was also considered a significant predictor of ECW (β = −0.02, *p* < 0.01 for females and β = −0.03, *p* < 0.01 for males) explaining alone 32% and 24% of ECW values, respectively (the unadjusted r^2^). When adjusted to covariables the R was considered a significant predictor in females (β = −0.03, *p* < 0.01) and males (β=−0.06, *p* < 0.01) of TBW values. R explained alone (without adjustment for covariables) about 39% in females and 51% in males.

As described in Table 5, Xc was considered a significant predictor of ECW (β = −0.06, *p* < 0.01 for females and β = −0.12, *p* < 0.01 for males) and is directly related to the ICW (β = 0.06, *p* = 0.069 for females and β = 0.08, *p* = 0.010 for males).

The Xc explains without adjustment for confounding variables 35% and 32% of ECW values and 9% and 14% of ICW values in females and males, respectively.

**Figure 1:**
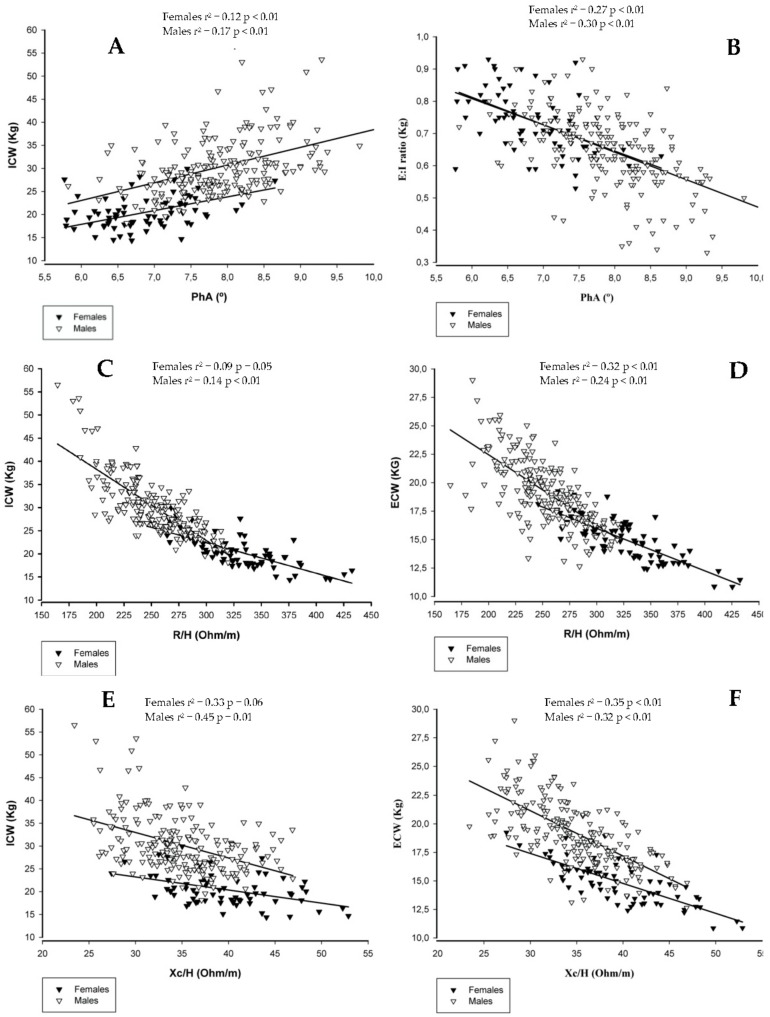
The independent association of BI parameters with fluid-related variables assessed through dilution techniques. Panel (**A**): PhA vs ICW; Panel (**B**): PhA vs E:I ratio; Panel (**C**): R/H vs ICW; Panel (**D**): R/H vs ECW; Panel (**E**): Xc/H vs ICW; Panel (**F**): Xc/H vs ECW. Abbreviations: PhA, phase angle; R/H, ratio between resistance and height (m); E:I ratio, ratio between extra-to-intracellular water; Xc/H, ratio between reactance and height (m); ECW, extracellular water; ICW, intracellular water.

The associations between BI parameters and fluid-related variables assessed through dilution techniques are displayed in Figure 1.

## 4. Discussion

The main findings of this study conducted in athletes included: (i) athletes with higher PhA values were those presenting higher values of TBW, particularly at the ICW compartment and, consequently, lower E:I ratio (representing body fluid distribution); (ii) higher Xc values predicted lower ECW and higher ICW; and (iii) R is strongly associated with water compartments. The role of these BI parameters on fluid compartments remained significant regardless of FM, FFM, age, height and sports category. The adjustment for the covariates was performed since FM, FFM, age, height [11], and sport category [26] were shown to have an impact on the variables under study.

In a recent study, a strong association between E:I ratio and PhA in healthy people was observed [11]. Few studies [4,17] explored associations between BI measurements and reference determinations of fluid volumes in athletes using tracer dilution methods. Marini and colleagues [17] confirmed in athletes that PhA detected E:I differences in both sexes, with lower PhA values in subjects with higher E:I ratio, independently of age and sports category. However, the authors did not adjust these associations for body composition. 

Regarding R, in this study, it was found that the males presented significantly lower values than the females (329.0Ω/m ± 39.6 and 251.8Ω/m ± 31.8, respectively). These values extend results observed in other studies performed in athletes [6,17]. R refers to the opposition offered by the human body to the passage of an electric current. In short, the conductivity of the tissues is proportional to the amount of body water and electrolytes [8]. Men presented significantly higher values of TBW than women, which means that the electric current flows more freely in the male body than in the female body. In addition, women presented higher FM values than men. FM has a poor conducting ability due to its low water constitution. In both sexes, as expected, the lowest R values are found in athletes who have higher TBW, ICW and ECW after adjusting for covariates. 

Reactance has been associated with cellular integrity. Theoretically, in biological conductors, high reactance values are expected from a BI measurement in healthy membranes with higher integrity. The healthy cell membranes act as capacitors by storing the current and releasing it. For example, it was found a Xc decrease after muscle injury (greater decline in major lesions) attributable to the muscle cell damage [12]. As a result, PhA also decreased. Thus, Xc is proportional to cell membrane integrity with Xc and PhA decrements occurring when the cell membrane is compromised. According to Liedtke [27], Xc is a measure of the volume of cell membrane capacitance and an indirect measure of the intracellular volume. In our study, higher Xc values explained higher ICW values in both sexes, regardless of height, FFM, FM, age and sports category.

In addition, we observed that Xc was a predictor of ECW values in males and females. Marini and colleagues [17] found similar results. The negative relationship between Xc and ECW in both sexes may be a possible biological justification for the high values of Xc in this study when compared to a clinical population [28,29]

Concerning PhA, in this study males presented a mean value of 7.9° ± 0.7 and females a mean value of 6.9° ± 0.6. These values are similar to those found in other studies conducted in athletes or active young population [6,17,24,30,31]. However, the values found in present study are slightly below those proposed by Barbosa-Silva and colleagues [32] according to gender and age for healthy adult population. As shown previously, men have higher PhA values than women [11,17,24,32], despite in the present study the Xc values were higher in females than in males (39.5 Ω/m ± 5.4 and 34.8 Ω/m ± 4.9, respectively), the lower R in the male group may be part of the explanation for a larger PhA in men relative to women. Indeed, PhA is a significant predictor of ICW in females and males, regardless of the covariates. It is sufficiently clear how important ICW is in increasing performance in athletes. For example, Silva and colleagues [2] observed in elite judo athletes that there is a decrease in the upper limbs power, in athletes that decreased the ICW compartment. In addition, ICW appears to be a good predictor of the risk of lower grip strength in elite judo athletes [3], essential for attack and defense techniques. It has long been postulated that a larger intracellular volume is an anabolic signal promoter according to "The Cell Swelling Theory" and consequent increase in cell volume. In fact, cell swelling leads to anabolism, whereas cell shrinkage promotes catabolism [33,34]. Considering the above-mentioned information, it seems plausible that PhA a predictor of ICW in athletes.

PhA was also considered a significant predictor of TBW in males. There is currently robust scientific evidence regarding the importance of TBW in biological performance, and only a small reduction in the hydration state may limit some of the body’s physiological processes, namely, power, muscular strength and endurance [2,35]. Although the amount of TBW is of extreme importance, focusing on this parameter alone can be limiting. Ribeiro and colleagues [30] in a study conducted in a non-athletic population, found that despite unchanged TBW, strength training contributed to a greater expansion of ICW, resulting in a reduction of the E:I ratio and an increased PhA. A better understanding of how TBW is distributed through its water compartments is required. 

The E:I ratio has previously been studied in a healthy non-athletic adult population [10] by using labeled tritium dilution (^3^H_2_O) to determine TBW, total body potassium for ICW and ECW estimated by the difference between TBW and ICW. The authors verified that E:I was considered a predictor of PhA. Our results extend the aforementioned findings as the PhA was considered a strong predictor of the E:I ratio in athletes independently of height, FM, FFM, age and sports category. 

Lower E:I values are often found in athletes. For example, Marini and colleagues [17] found E:I values of 0.6 ± 0.1 in men and 0.7 ± 0.1 in women. On the other hand, higher values were found in a study by Gonzales and colleagues [11] conducting in healthy non-athletic adults. These authors found E:I values of 1.03 in women and 0.79 in men. We can speculate that athletes have well-defined water regulation mechanisms at the level of cell membranes preserving a greater amount of ICW. 

In Marini’s study the raw parameters of the BIA were evaluated by means of a single BIA frequency (BIA 101 Anniversary, Akern, Florence, Italy). This method utilizes a phase-sensitive impedance that introduces a constant, low-level alternating current, enabling direct measurement of PhA and Z and then R and Xc can be calculated. In our study BIS was used to measure resistance R and reactance Xc while impedance and PhA were calculated at each measured frequency (in this case 50kHz which is reported as a standard constant current condition, commonly used by most studies). In a recent study by Silva and colleagues [25], a comparison between single and multi-frequency devices (Akern and BIS) was conducted in a highly active population. The authors found that the both devices were highly related in measuring raw BIA parameters at a frequency of 50kHz but a lack of agreement was observed at the individual level. In the aforementioned study, the BIS instrument provided significantly lower values of R and Xc but higher values of PhA at 50kHz. 

Thus, we should address as a limitation of this study the fact that the use of a non-phase sensitivity BIA equipment may not be free of a latent model error prediction in the calculation of PhA, though reference techniques were used to determine water and its compartments. In addition, this study presented a cross-sectional design limiting the inference for longitudinal relationships. Thus, further studies should be carried out using a longitudinal approach to explore how raw BIA measures impact fluid-related changes, especially in light of the high physical damage resulting from a large volume of training and competitions during the sport season.

In conclusion, athletes with higher values of PhA have a lower value of E:I ratio and a higher value of ICW and TBW, regardless of height, body composition, sports category, and age. As expected, R values were predictors of TBW, ECW and ICW. Lower R values are observed in athletes who present higher TBW and its extra and intracellular water compartments. In both sexes, higher values of Xc are associated with a lower ECW expansion and with a higher ICW pool. The practical applicability of this study is clear for coaches, physicians, nutritionists, and exercise physiologists: new methods to assess hydration status that are time-efficient, simple, safe, precise, accurate and reliable are needed and the usefulness of raw BI parameters in the assessment water compartments and fluid distribution of elite athletes was highlighted in this study. 

## Figures and Tables

**Table 1:** Body composition, water compartments and raw bioelectrical impedance (BI) parameters at 50 kHz frequency.

Variable	WOMEN (N = 71)			MEN (N = 202)		
	Minimum	Mean ± SD	Maximum	Minimum	Mean ± SD	Maximum
**Age(y)**	15	20.4 ± 5.2	35	15	21.5 ± 4.5	38
**Body Mass (kg)**	48.5	63.9 ± 8.8	81.9	56.5	77.6 ± 13.0 ^§^	130.3
**Height (cm)**	152.2	170.8 ± 8.0	195.0	164.4	181.4 ± 9.2 ^§^	204.7
**BMI (kg/m^2^)**	17.7	21.9± 2.1	26.0	18.6	23.7 ± 4.1 ^§^	58.7
**FM (kg)**	8.3	15.6 ± 4.3	26.5	4.8	11.2 ± 5.8 ^§^	45.2
**FFM (kg)**	35.4	48.0 ± 6.1	60.4	49.5	65.6 ± 8.7 ^§^	91.7
**Resistance (Ω)**	435.1	561.2 ± 65.9	720.1	305.6	456.0 ± 56.5 ^§^	582.1
**Reactance (Ω)**	48.8	67.3 ± 8.5	87.1	43.5	62.9 ± 7.9 ^§^	85.2
**Impedance (Ω)**	438.1	565.1 ± 68.3	724.4	120.6	459.5 ± 62.1 ^§^	587.9
**R/H ((Ω/m)**	246.0	329.0 ± 39.6	432.5	164.6	251.8 ± 31.8 ^§^	319.7
**Xc/H (Ω/m)**	27.4	39.5 ± 5.4	52.9	23.4	34.8 ± 4.9 ^§^	46.9
**Z/H (Ω/m)**	247.7	331.4 ± 39.9	435.7	166.3	254.5 ± 32.2 ^§^	321.9
**Phase Angle (°)**	5.8	6.9 ± 0.6	8.7	5.8	7.9 ± 0.7 ^§^	10.0
**Intracellular Water (kg)**	14.4	20.5 ± 3.5	29.9	18.1	30.3 ± 6.2 ^§^	56.5
**Extracellular Water (kg)**	11.0	14.9 ± 1.9	19.25	12.7	19.3 ± 2.9 ^§^	29.0
**Total Body Water (kg)**	25.6	35.4 ± 4.9	45.9	34.5	49.5 ± 7.8 ^§^	76.3
**ECW/ICW ratio**	0.5	0.7 ± 0.1	0.9	0.3	0.6 ± 0.1 ^§^	0.9

Abbreviations: BMI, body-mass index; FM, fat mass; FFM, free fat mass; E:I, extracellular:intracellular water ratio; R/H, resistance standardized for height; Xc/H, reactance standardized for height; Z/H, vector length standardized for height; SD, standard deviation. ^§^ *P* < 0.01 (independent sample *t* test).

**Table 2:** Pearson correlation coefficients (r) between raw bioelectrical impedance parameters and fluid volumes and distribution.

Variable	ICW			ECW			TBW			E:I		
	Total N = 273	Women N = 71	Men N = 202	Total N = 273	Women N = 71	Men N = 202	Total N = 273	Women N = 71	Men N = 202	Total N = 273	Women N = 71	Men N = 202
**PhA°**	0.60 ^**^ p < 0.01	0.34 ^**^ p = 0.003	0.42 ^**^ p < 0.01	0.25 ^**^ p < 0.01	−0.08 *p* = 0.505	−0.10 *p* = 0.152	0.53 ^**^ p < 0.01	0.22 *p* = 0.071	0.30 ^**^ p < 0.01	−0.61 ^**^ p < 0.01	−0.52 ^**^ p < 0.01	−0.55 ^**^ p < 0.01
**R/H (Ω/m)**	−0.84 ^**^ p < 0.01	−0.75 ^**^ p < 0.01	−0.78 ^**^ p < 0.01	−0.81 ^**^ p < 0.01	−0.79 ^**^ p < 0.01	−0.69 ^**^ p < 0.01	−0.91 ^**^ p < 0.01	−0.84 ^**^ p < 0.01	−0.87 ^**^ p < 0.01	0.40 ^**^ p < 0.01	0.19 *p* = 0.110	0.27 ^**^ p = 0.001
**Xc/H (Ω/m)**	−0.55 ^**^ p < 0.01	−0.44 ^**^ p < 0.01	−0.44 ^**^ p < 0.01	−0.74 ^**^ p < 0.01	−0.76 ^**^ p < 0.01	−0.68 ^**^ p < 0.01	−0.66 ^**^ p < 0.01	−0.61 ^**^ p < 0.01	−0.60 ^**^ p < 0.01	0.01 *p* =0.148	−0.17 *p* = 0.157	−0.12 *p* = 0.089
**FFM**	0.89 ^**^ p < 0.01	0.82 ^**^ p < 0.01	0.81 ^**^ p < 0.01	0.84 ^**^ p < 0.01	0.87 ^**^ p < 0.01	0.72 ^**^ p < 0.01	0.95 ^**^ p < 0.01	0.92 ^**^ p < 0.01	0.91 ^**^ p < 0.01	−0.41 ^**^ p < 0.01	−0.20 ^**^ p < 0.01	−0.27 ^**^ p < 0.01
**FM**	0.20 ^**^ p < 0.01	0.27 ^*^ p = 0.03	0.59 ^**^ p < 0.01	0.16 ^**^ p < 0.01	0.55 ^**^ p < 0.01	0.46 ^**^ p < 0.01	0.21 ^**^ p < 0.01	0.40 ^**^ p < 0.01	0.64 ^**^ p < 0.01	−0.01 *p* = 0.95	0.21 *p* = 0.09	−0.21 ^**^ p < 0.01

Abbreviations: ICW, intracellular water; ECW, extracellular water; TBW, total body water; E:I, extracellular:intracellular water; Z/H, impedance standardized for height (m); Xc/H, reactance standardized for height (m); R/H, resistance standardized for height; PhA, phase angle. ^**^ Correlations were significant at *p* < 0.05; ^*^ correlations were significant at *p* < 0.01.

**Table 3:** Linear and Multiple Regression Analysis Between PhA and ICW, TBW and E:I.

ICW				TBW				E:I			
Women	β	SE	*p*	Women	β	SE	*p*	Women	β	SE	*p*
**PhA (°)**	1.62	0.38	<0.01	**PhA (°)**	1.25	0.38	0.02	**PhA (°)**	−0.08	0.02	<0.01
**Height (cm)**	−0.04	0.04	0.427	**Height (cm)**	−0.04	0.04	0.355	**Height (cm)**	0.001	0.002	0.792
**Sports Category**	0.27	0.29	0.354	**Sports Category**	−0.03	0.29	0.929	**Sports Category**	−0.02	0.01	0.145
**FFM (kg)**	0.52	0.06	<0.01	**FFM (kg)**	0.76	0.06	<0.01	**FFM (kg)**	−0.01	0.003	0.047
**FM (kg)**	0.01	0.06	0.984	**FM (kg)**	0.11	0.06	0.062	**FM (kg)**	0.01	0.003	0.045
**Age**	−0.12	0.04	0.009	**Age**	−0.11	0.04	0.011	**Age**	0.004	0.002	0.038
**Men**	**β**	**SE**	***p***	**Men**	**β**	**SE**	***p***	**Men**	**β**	**SE**	***p***
**PhA (°)**	2.70	0.33	<0.01	**PhA (°)**	2.01	0.29	<0.01	**PhA (°)**	−0.08	0.01	<0.01
**Height (cm)**	−0.06	0.03	0.082	**Height (cm)**	−0.07	0.03	0.017	**Height (cm)**	0.001	0.001	0.640
**Sports Category**	−0.03	0.30	0.912	**Sports Category**	0.51	0.27	0.061	**Sports Category**	0.01	0.01	0.205
**FFM (kg)**	0.54	0.04	<0.01	**FFM (kg)**	0.76	0.03	<0.01	**FFM (kg)**	−0.002	0.001	0.052
**FM (kg)**	0.18	0.04	<0.01	**FM (kg)**	0.20	0.04	<0.01	**FM (kg)**	−0.002	0.001	0.204
**Age**	−0.08	0.05	0.080	**Age**	−0.11	0.04	0.008	**Age**	0.001	0.001	0.844

Model adjusted for height, sports category, fat free mass, fat mass, and age. Abbreviations: PhA, phase angle; TBW, total-body water; ECW, extracellular water; ICW, intracellular water; FM; fat mass; FFM, fat-free mass; SE, standard error, *β*, regression coefficient.

**Table 4:** Multiple regression analysis between R and ICW, ECW and TBW.

ICW				ECW				TBW			
Women	β	SE	*p*	Women	β	SE	*p*	Women	β	SE	*p*
**R (Ω)**	−0.01	0.01	0.053	**R (Ω)**	−0.02	0.003	<0.01	**R (Ω)**	−0.03	0.01	<0.01
**Height (cm)**	0.01	0.07	0.887	**Height (cm)**	0.12	0.03	<0.01	**Height (cm)**	0.13	0.06	0.037
**Sports Category**	0.37	0.32	0.248	**Sports Category**	−0.17	0.12	0.154	**Sports Category**	0.20	0.27	0.462
**FFM (kg)**	0.42	0.11	<0.01	**FFM (kg)**	0.04	0.04	0.307	**FFM (kg)**	0.47	0.09	<0.01
**FM (kg)**	−0.06	0.06	0.343	**FM (kg)**	0.12	0.02	<0.01	**FM (kg)**	0.06	0.05	0.28
**Age**	−0.11	0.05	0.021	**Age**	0.01	0.02	0.740	**Age**	−0.11	0.04	0.01
**Men**	**β**	**SE**	***p***	**Men**	**β**	**SE**	***p***	**Men**	**β**	**SE**	***p***
**R (Ω)**	−0.03	0.01	<0.01	**R (Ω)**	−0.03	0.004	<0.01	**R (Ω)**	−0.06	0.01	<0.01
**Height (cm)**	−0.05	0.05	0.340	**Height (cm)**	0.17	0.03	<0.01	**Height (cm)**	0.12	0.04	0.001
**Sports Category**	−0.04	0.34	0.898	**Sports Category**	0.29	0.19	0.127	**Sports Category**	0.25	0.24	0.287
**FFM (kg)**	0.45	0.07	<0.01	**FFM (kg)**	−0.04	0.04	0.905	**FFM (kg)**	0.44	0.05	<0.01
**FM (kg)**	0.18	0.05	<0.01	**FM (kg)**	0.05	0.03	0.089	**FM (kg)**	0.23	0.03	<0.01
**Age**	−0.06	0.05	0.238	**Age**	−0.02	0.03	0.568	**Age**	−0.08	0.04	0.032

Model adjusted for height, sports category, fat free mass, fat mass, and age Abbreviations: R, resistance; TBW, total-body water; ECW, extracellular water; ICW, intracellular water; FM; fat mass; FFM, fat-free mass; SE, standard error, *β*, regression coefficient.

**Table 5:** Linear and multiple regression analysis between Xc and ICW, ECW and TBW.

ICW				ECW				TBW			
Women	β	SE	*p*	Women	β	SE	*p*	Women	β	SE	*p*
**Xc(Ω)**	0.06	0.03	0.069	**Xc(Ω)**	-0.06	0.01	<0.01	**Xc(Ω)**	0.001	0.03	0.968
**Height (cm)**	−0.13	0.05	0.012	**Height (cm)**	0.04	0.02	0.044	**Height (cm)**	−0.09	0.05	0.071
**Sports Category**	0.20	0.32	0.526	**Sports Category**	−0.24	0.12	0.062	**Sports Category**	−0.03	0.31	0.916
**FFM(kg)**	0.66	0.07	<0.01	**FFM(kg)**	0.17	0.03	<0.01	**FFM (kg)**	0.83	0.07	<0.01
**FM (kg)**	−0.03	0.06	0.645	**FM (kg)**	0.10	0.03	<0.01	**FM (kg)**	0.07	0.06	0.286
**Age**	−0.12	0.05	0.017	**Age**	0.003	0.02	0.859	**Age**	−0.114	0.05	0.017
**Men**	**β**	**SE**	***p***	**Men**	**β**	**SE**	***p***	**Men**	**β**	**SE**	***p***
**Xc(Ω)**	0.08	0.03	0.010	**Xc(Ω)**	−0.12	0.02	<0.01	**Xc(Ω)**	−0.03	0.03	0.306
**Height (cm)**	−0.24	0.03	<0.01	**Height (cm)**	0.06	0.02	0.003	**Height (cm)**	−0.18	0.03	<0.01
**Sports Category**	0.20	0.34	0.562	**Sports Category**	0.45	0.19	0.020	**Sports Category**	0.65	0.30	0.031
**FFM (kg)**	0.71	0.05	<0.01	**FFM (kg)**	0.12	0.03	<0.01	**FFM (kg)**	0.83	0.04	<0.01
**FM (kg)**	0.16	0.05	0.002	**FM (kg)**	0.03	0.03	0.294	**FM (kg)**	0.19	0.04	<0.01
**Age**	−0.08	0.05	0.091	**Age**	−0.02	0.03	0.611	**Age**	−0.10	0.05	0.025

Model adjusted for height, sports category, fat free mass, fat mass, and age; Abbreviations: Xc, reactance; TBW, total-body water, ECW, extracellular water; ICW, intracellular water; FM; fat mass; FFM, fat-free mass; SE, standard error, β, regression coefficient.
